# Thermodynamic State Machine Network

**DOI:** 10.3390/e24060744

**Published:** 2022-05-24

**Authors:** Todd Hylton

**Affiliations:** Department of Mechanical and Aerospace Engineering, University of California, San Diego, CA 92093, USA; thylton@ucsd.edu

**Keywords:** thermodynamic computing, thermodynamicalism, machine learning, scale integration, input functionalization, active equilibration

## Abstract

We describe a model system—a thermodynamic state machine network—comprising a network of probabilistic, stateful automata that equilibrate according to Boltzmann statistics, exchange codes over unweighted bi-directional edges, update a state transition memory to learn transitions between network ground states, and minimize an action associated with fluctuation trajectories. The model is grounded in four postulates concerning self-organizing, open thermodynamic systems—transport-driven self-organization, scale-integration, input-functionalization, and active equilibration. After sufficient exposure to periodically changing inputs, a diffusive-to-mechanistic phase transition emerges in the network dynamics. The evolved networks show spatial and temporal structures that look much like spiking neural networks, although no such structures were incorporated into the model. Our main contribution is the articulation of the postulates, the development of a thermodynamically motivated methodology addressing them, and the resulting phase transition. As with other machine learning methods, the model is limited by its scalability, generality, and temporality. We use limitations to motivate the development of thermodynamic computers—engineered, thermodynamically self-organizing systems—and comment on efforts to realize them in the context of this work. We offer a different philosophical perspective, thermodynamicalism, addressing the limitations of the model and machine learning in general.

## 1. Introduction

The remarkable progress in machine learning systems over the past decade has led to an equally remarkable increase in the computational resources required to train them. One recent report indicates that the number of computing resources used to train state-of-the-art, large scale models has doubled every 3.4 months since 2012, as compared to a more traditional 2-year doubling rate prior to 2012 [1]. Such trends suggest the consideration of fundamentally new approaches to machine learning. We have previously outlined the vision for such an approach, *thermodynamic computing* [2,3,4], which is motivated by the hypothesis that the ubiquitous self-organization of the natural world is fundamentally thermodynamic [5,6], and that this capacity can be incorporated into computing hardware. In this work, we further articulate this hypothesis as four postulates concerning self-organizing, open thermodynamic systems, and build a model system—the *thermodynamic state machine network* (TSMN)—reflecting these postulates.

The TSMN is a machine learning model comprising a network of probabilistic, stateful automata that equilibrate according to Boltzmann statistics, exchange codes over unweighted bi-directional edges, update a state transition memory to learn transitions between network ground states, and minimize an action associated with fluctuation trajectories. The dynamics of the TSMN are governed by particle-like excitations whose motion is easily visualized as videos. Unlike neural network-based machine learning models, which learn long-term memory in weighted edges connecting nodes, the TSMN learns long-term memory in state transition memories at each node. After sufficient exposure to periodically changing inputs, a diffusive-to-mechanistic phase transition emerges in the network dynamics, and the evolved networks show spatial and temporal structures that look like a spiking neural network, although no such structures were incorporated into the model. Before the phase transition, the dynamics of the TSMN are diffusive, difficult to compute, and difficult to describe using an algorithm; after the transition, the dynamics are mechanistic, easy to compute, and easy to describe using an algorithm. Like other machine learning models, the TSMN is limited in its ability to generalize, scale, and represent temporal processes. We use these limitations as motivation for the development of thermodynamic computers. We also discuss several published experimental efforts that address these motivations and compare them to the postulates used to develop the TSMN.

The TSMN is inspired by theories of open thermodynamic systems. The non-equilibrium fluctuation theorems of Jarzynski [7], and Crooks [8] and Still’s “thermodynamics of prediction” [9], are the conceptual framework for the equilibration (Section 2.3) and adaptation (Section 2.5) methodologies of the TSMN. Ideas on “active inference” [10] influenced the inferencing methodology (Section 2.6), ideas on “dissipative adaptation” [11] influenced the adaptation methodology (Section 2.5), and ideas on “causal entropic forces” [12] influenced the state update methodology (Section 2.4). The TSMN is also inspired by other thermodynamically grounded model systems, such as Ising models [13], Hopfield networks [14], and Boltzmann machines [15], but is distinguished from them by the details of its composition (e.g., code-transport, divergence-less node states, unweighted edges, and state transition memory) and learning methodology. Like Ising models and related neural network models, the TSMN is a distributed collection of autonomous nodes that collectively self-organize through local interactions, but the TSMN is different from these models in that it has a large degeneracy of stable ground states separated by particle-like excitations. The ground state degeneracy means that there is more than one “solution” to any “problem” posed by external inputs, and that the network is generally not frustrated. Other influences on this work include “physical reservoir computers” [16] and related model systems such as “liquid state machines” [17] and “echo state networks” [18]. The TSMN shares with these approaches the goal of learning and predicting time-varying inputs, but the TSMN integrates this ability in a single, recurrent, adaptive architecture. A machine-learning method called “equilibrium propagation” [19] employs similar ideas as the TSMN but uses different methodologies and addresses neural network models. The TSMN also builds upon ideas and methods from our earlier work on a “thermodynamic neural network” model [20].

Our contributions in this article include (1) an articulation of postulates concerning the self-organization of complex, open, thermodynamic systems, (2) a new network model and machine learning methodology for state machines addressing these postulates, (3) a demonstration of a phase change in the dynamics of the TSMN when exposed to periodic inputs, and (4) a consideration of classical and thermodynamic computing in the context of the TSMN and its limitations. We hope as well that this work serves as a pedagogical example of the emergence of computation from a thermodynamic, evolutionary process.

The paper is organized as follows. In Methods (Section 2) we summarize the model approach, describe the Postulates (Section 2.1) that motivated its development, detail the model equilibration and adaptation methodologies (Section 2.2, Section 2.3, Section 2.4, Section 2.5 and Section 2.6), and describe the Visualization Section 2.7 of the model dynamics. In Results (Section 3) we show and analyze the dynamics of networks without (Section 3.1) and with (Section 3.2) state transition memory. In Discussion (Section 4) we discuss the limitations of the model (Section 4.1) and use these limitations to motivate Thermodynamic Computing (Section 4.2) and to compare related experimental efforts, offer an interpretation of Classical Computing (Section 4.3), and speculate on a different philosophical perspective called Thermodynamicalism (Section 4.4). As an appendix, we offer a conceptual circuit model for the TSMN (Appendix A: TSMN Circuit Model).

## 2. Methods

**Figure 1:**
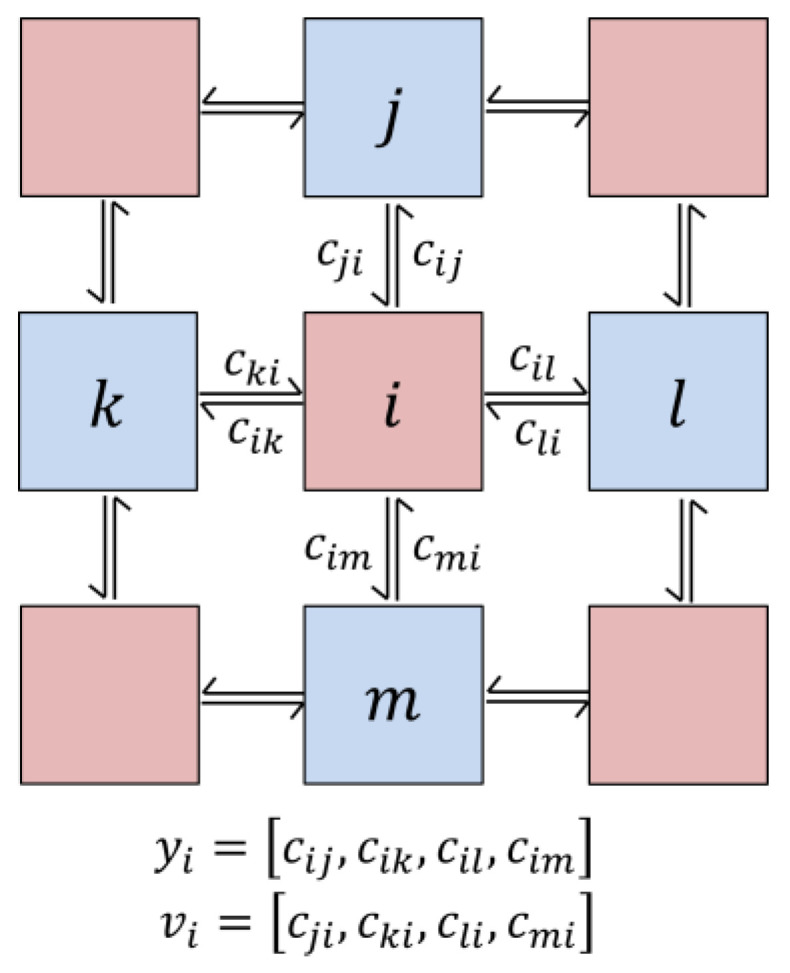
*Network structure, edge codes, node outputs and inputs*. Nodes i and j exchange codes $c_{ij}$ and $c_{ji}$. The output $y_{i}$/input $v_{i}$ of node i is the vector of its output/input edge codes. In sampling the network evolution, the network is bi-partitioned in a checker-board pattern (salmon and blue nodes). The inputs of one partition are the outputs of the other partition.

The TSMN is a bi-directional, unweighted network of probabilistic, stateful automata. Figure 1 illustrates the basic network structure. We call $c_{ij}$ the *edge code* sent from node i to node j. The collection of edge codes from node i define its output $y_{i}$, while the collection of edge codes to node i define its input $v_{i}$. $c_{ij}$ “interacts” with edge code $c_{ji}$ sent from node j to node i along the same i↔j edge, such that edges with complementary codes have low energy and edges with like codes have high energy. The TSMN can be thought of a kind of “Ising model” in which the interaction of the nodes is motivated by the transportation of codes (or “charges”) through them. To effectively “move” these codes, the node must configure its output to avoid a “build up” of like codes on its edges. Although more complex networks are possible, here we consider only 2-dimensional, 4-nearest-neighbor networks and binary edge codes $c_{ij}\in \left\{0,1\right\}$ because the dynamics are easy to visualize.

This section is organized as follows. Postulates (Section 2.1) explains the assumptions behind the methods described in this section. A Node Interaction Model (Section 2.2) includes internal nodes, which conserve and transport edge codes, and external nodes, which represent inputs from environmental potentials and act as sources and sinks of edge codes to which the network must respond. An edge code interaction energy is defined such that network configurations that more effectively transport edge codes have lower energy. In simulation, the network is repeatedly sampled using a Large-Spatial-Scale, Short-Timescale Equilibration Algorithm (Section 2.3). The node interaction model is then augmented with a State Transition Memory (Section 2.4), which is accessed using a state-indexing method, and incrementally updated with every network ground state transition using a Small-Spatial-Scale, Long-Timescale Adaptation Algorithm (Section 2.5) that minimizes the free energy of the network. A method for Inference of Dormant Inputs/Generation of Outputs (Section 2.6) is also described. Methods for visualizing the dynamics implied by Section 2.2, Section 2.3, Section 2.4, Section 2.5 and Section 2.6 are described in Visualization (Section 2.7).

### 2.1. Postulates

**Figure 2:**
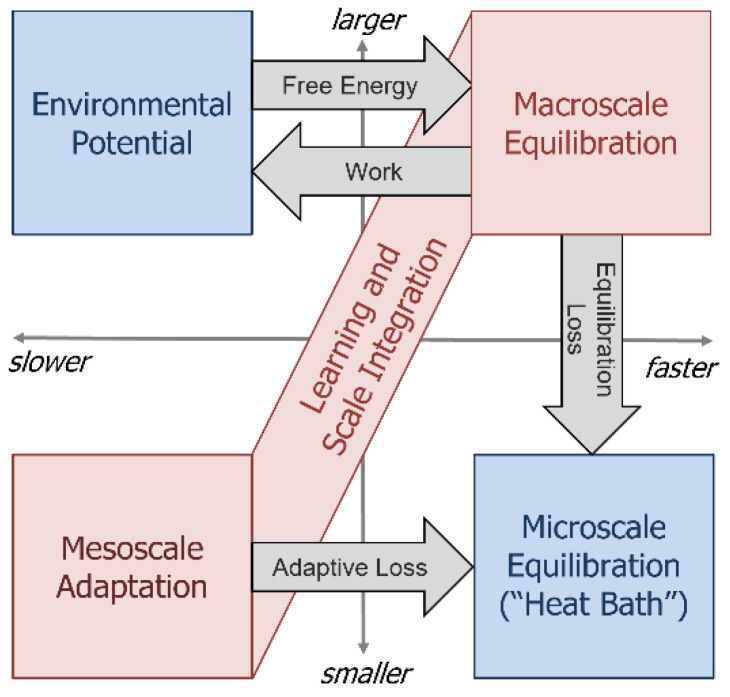
*Illustration of postulates*. An open thermodynamic system (red) equilibrates with an environment (blue) that provides a source of potential (**upper left**) and a reservoir of small-fast excitations, or “heat bath” (**lower right**). The open system comprises the ability to equilibrate rapidly at a large scale (**upper right**) and to adapt slowly at a small scale (**lower left**), integrated by a learning process. To maintain homeostasis, conserved quantities (e.g., energy) taken from the environment by the open system must be returned to it, which drives the learning process and integrates scales. If the open system has functionalized input potentials from the environment, then it may also interact with the environment by doing work on it to “retrieve” missing input potentials. The ability to do work on the environment implies that the open system stores energy, and that its equilibration is active.

We have previously integrated the thermodynamic concepts of conservation, potentiation, fluctuation, dissipation, and equilibration to create a thermodynamic neural network model [20]. In this section we articulate the result of this integration as a set of postulates concerning self-organizing, complex, open thermodynamic systems (see Figure 2). We will use these postulates to motivate the detailed methodologies of the following sections.

Scale-integration—The integrity of an open system depends on its ability to rapidly change its internal, small-scale organization to address the external, large-scale changes of the potentials in its environment. We call this alignment of large- and small-scale features *scale-integration*, and the process of creating it is referred to as *learning*. In natural systems (e.g., organisms), we suppose that scale-integration is a natural consequence of multi-scale equilibration between an open system and its environment. In the TSMN, however, scale integration is created using a two-piece equilibration algorithm—one piece providing large-spatial-scale, short-timescale equilibration with an environment (Section 2.3), and another piece providing small-spatial-scale, long-time scale internal parameter adaptation (Section 2.5) that facilitates future equilibration. A familiar example of such two-piece methodology is a forward-pass through an artificial neural network providing the large-spatial-scale, short-timescale equilibration (measured as output “errors”) with a training data set, and a backward-pass providing the small-spatial-scale, large-timescale internal adaptation (typically as “weight updates”) to reduce future output/equilibration errors.

Transport-Driven Self-Organization—Any open system in homeostasis with its environment must return to the environment any conserved quantity that is taken from it. Imbalances in the distribution of conserved quantities in the environment create thermodynamic potentials (free energy) that can drive their transport through the open system, thereby relieving those potentials and creating entropy in the environment. Sustained entropy creation favors the evolution of organization within the open system that facilitates the transport of these conserved quantities, while also stabilizing the associated organization.

Input Functionalization—If an open thermodynamic system evolves stable internal organizations that efficiently transport conserved quantities in response to external input potentials, then we say that the system has “functionalized” its inputs. Input functionalization enables the open system to “infer” or “predict” the existence of potentials that are missing in the current context but were present during the learning process. For example, a naïve artificial neural network is first trained on stimuli-label pairs (the input potentials before functionalization) to develop its internal organization, such that the trained network can subsequently infer labels (the missing input potentials after functionalization) from stimuli (the input potentials available after functionalization). In some cases, the missing, inferred potentials are realized as “outputs” that prompt the environment to provide additional inputs. A recursive exchange of such inputs and outputs is frequently called an “interaction”. For example, an artificial neural network may be used to provide product recommendations in response to a query by an online shopper. The recommendation may prompt the shopper to provide additional input that may be used to provide a new recommendation, and to improve training of the recommending system.

Active Equilibration—The capacity of an open system to interact with an environment after it has functionalized its inputs requires that it store energy internally, i.e., that the outputs supplied to its environment are themselves an aggregation of conserved quantities constituting a thermodynamic potential to which the environment must respond. Similarly, the ability of an open system to proactively change its internal organization in response to changes in the potentials in its environment requires that it store energy. We call this ability to store and use energy to interact with the environment to maintain homeostasis and to proactively change internal organization *active equilibration*.

### 2.2. Node Interaction Model

To implement the idea of transport driven self-organization, internal nodes are restricted to have “non-divergent” outputs: i.e., $y_{i}$ are restricted to having equivalent numbers of 0 s and 1 s. Of the $2^{4}=16$ possible outputs for a node with binary edge codes and connections to four nearest neighbors, only 6 outputs satisfy this constraint. In particular, any internal node output $y_{i}$ must be drawn from the set $\gamma_{i}$, where

(1) $$\gamma_{i}=\left\{\left[1,1,0,0\right],\left[1,0,1,0\right],\left[1,0,0,1\right],\left[0,0,1,1\right],\left[0,1,0,1\right],\left[0,1,1,0\right]\right\},\;\;y_{i}\in \gamma_{i}.$$

We note that the assumption of unweighted, unsigned edges, and the requirement of non-divergent node outputs in the TSMN, excludes the use of binary node states commonly found in Ising and neural network models.

External nodes source and sink codes to the network and are defined as having maximal divergence. The corresponding output set is

(2) $$\gamma_{i}^{ext}=\left\{\left[0,0,0,0\right],\left[1,1,1,1\right]\right\},\;\;y_{i}^{ext}\in \gamma_{i}^{ext}.$$

In the examples that follow, we constrain the use of external nodes such that their aggregate output is non-divergent. Hence, for every external node with output [0,0,0,0], there is a complementary external node with output [1,1,1,1].

We define an edge interaction energy matrix $K_{ij}$ as

(3) $$K_{ij}=\left(\delta_{c_{ij},c_{ji}}-1\right)=-\delta_{c_{ij},{\bar{c}}_{ji}},$$

where δ is the Kronecker delta function and ${\bar{c}}_{ji}$ is the complementary code to $c_{ji}$. By this definition, the edge interaction energy is low ($K_{ij}=-1$) when the edge codes are different, and high ($K_{ij}=0$) when the edge codes are identical. We call edges with identical codes *edge-excitations*. The energy $H_{i}$ of node i is the sum over the energy of its edges.

(4) $$H_{i}=\underset{j\in i_{nn}}{\sum }K_{ij},$$

where $i_{nn}$ is the set of indices to the nearest neighbors of node i. A node achieves its lowest energy configuration when its output edge codes complement its input edge codes on every edge, ${\bar{c}}_{i_{nn},i}=c_{i,i_{nn}}$, a condition that can be met only if its inputs also have no divergence (i.e., $v_{i}\in \gamma_{i}$). The energy ε of the network is the sum over the internal node energies,

(5) $$\varepsilon =\overset{n}{\underset{i=1}{\sum }}H_{i},$$

where n is the number of internal nodes. A ground state of the network is obtained when the codes are complemented on every edge, a non-trivial requirement given the constraints of $\gamma_{i}$. Given the non-divergence of the internal nodes and maximal divergence of the external nodes, low-energy network states can be thought of as “transporting” codes between complementary external nodes. We note that the network ground states are highly degenerate, which we will show to be an important feature in the network’s ability to respond to inputs from external nodes and to learn a state transition memory. From the perspective of automata theory, an internal node “accepts” inputs from its connected nodes when those inputs allow a node ground state, and the network as a whole “accepts” inputs from its external nodes when those inputs allow a network ground state. In Section 2.5 we shall use these acceptance criteria as the keys to drive learning within the network, and in Section 4.3 to discuss connections to classical computing.

### 2.3. Large-Spatial-Scale, Short-Timescale, Equilibration Algorithm

The probability P(y) of a particular network output state $y=\left(y_{1},y_{2}\ldots y_{n}\right)$ is assumed to be governed by a Boltzmann distribution at inverse temperature β and with partition function Z as

(6) $$P\left(y\right)=\frac{1}{Z}\cdot exp\left(-\beta \varepsilon \left(y\right)\right)=\frac{1}{Z}\cdot \underset{i}{\prod }exp\left(-\beta H_{i}\left(y_{i}\right)\right)\;Z=\underset{y\in \gamma }{\sum }exp\left(-\beta \varepsilon \left(y\right)\right)=\underset{y\in \gamma }{\sum }\underset{i}{\prod }exp\left(-\beta H_{i}\left(y_{i}\right)\right)=\underset{i}{\prod }\underset{y_{i}\in \gamma_{i}}{\sum }exp\left(-\beta H_{i}\left(y_{i}\right)\right),$$

where $\gamma =\left(\gamma_{1},\gamma_{2}\ldots \gamma_{n}\right)$ is the set of all possible node outputs. We employ a Markov Chain Monte Carlo (MCMC)/Gibbs Sampling [21] algorithm that repeatedly samples Equation (6) to generate the large-spatial-scale, short-timescale dynamics of the TSMN. The algorithm partitions the nodes into two groups in a checkerboard pattern according to their placement in the 2D network, such that the inputs to the nodes in one partition are the outputs from the nodes in the opposite partition (Figure 1). We sample the two network partitions alternately by holding the node outputs in one partition fixed, while sampling the node outputs of the other partition. In this way, each node output can be sampled independently using Equation (7).

(7) $$P\left(y_{i}|v_{i}\right)=\frac{1}{Z_{i}}exp\left(-\beta H_{i}\left(y_{i}|v_{i}\right)\right)\;Z_{i}=\underset{y_{i}\in \gamma_{i}}{\sum }exp\left(-\beta H_{i}\left(y_{i}|v_{i}\right)\right)$$

We refer to the evolution of the network as we repeatedly sample the partitions as its “equilibration dynamics”. In the videos of the equilibration dynamics in Section 3, each frame in the video represents the update of the nodes in one partition.

### 2.4. State Transition Memory

We augment the node interaction energy $H_{i}$ of each internal node with a state transition energy $T_{i}$ and a weighting parameter σ, such that the total energy $E_{i}$ of node i becomes

(8) $$E_{i}\left(y_{i}|v_{i},s_{i}\right)=H_{i}\left(y_{i}|v_{i}\right)-\sigma \cdot T_{i}\left(y_{i}|s_{i}\right).$$

where $s_{i}$ is an index specifying the node state, which we define as a temporal sequence or “stack” of the last d outputs of node i at *a network ground state*. If we label the node output associated with the most recent network ground state as ${\tilde{y}}_{i}^{1}$, the next most recent as ${\tilde{y}}_{i}^{2}$, etc., then

(9) $$s_{i}=\left[{\tilde{y}}_{i}^{1},{\tilde{y}}_{i}^{2}\ldots {\tilde{y}}_{i}^{d}\right].$$

We refer to d as the “depth” of the state-index. Every time the network reaches a new ground state, each node updates its state-index by pushing its current output onto the top of the stack and popping the oldest value off the bottom the stack. Hence, state-index updates at each node are synchronized across the network because they only occur at network (not node) ground states. Network dynamics between network ground states can be thought of as *fluctuations*, which explore the network output space but do not affect the state of the network. The node output distribution of Equation (7) used by the MCMC algorithm is modified as

(10) $$P\left(y_{i}|v_{i},s_{i}\right)=\frac{1}{Z_{i}}exp\left(-\beta E_{i}\left(y_{i}|v_{i},s_{i}\right)\right)\;Z_{i}=\underset{y_{i}\in \gamma_{i}}{\sum }exp\left(-\beta E_{i}\left(y_{i}|v_{i},s_{i}\right)\right),$$

We note that $E_{i}$ comprises a component $H_{i}$, which is fundamentally spatial, and a component $T_{i}$, which is fundamentally temporal. The addition of $T_{i}$ also confers upon the TSMN a “state-energy landscape”: the idea that a change in state modifies the energy landscape and that a change in the energy landscape modifies the state in an iterative process that breaks time-reversal symmetry.

### 2.5. Small-Spatial-Scale, Long-Timescale, Adaptation Algorithm

To construct the small-spatial-scale, long-timescale adaptation algorithm, we extend the single-step (i.e., short-timescale) network energy ε of Equation (5) into multi-step (i.e., long-timescale) network action A as,

(11) $$A\left(y^{0}\to y^{m}\right)=\overset{y^{m}}{\underset{y=y^{0}}{\sum }}\varepsilon \left(y\right),$$

which is the sum of the network energies over a sequence of network updates where $y^{0}\to y^{m}\equiv \left[y^{0},y^{1}\ldots y^{m}\right]$ indicates a particular m-step *trajectory* between network outputs $y^{0}$ and $y^{m}$. We weight the probability of these trajectories using a Boltzmann distribution as

(12) $$P\left(y^{0}\to y^{m}\right)=\frac{1}{Z}\cdot exp\left(-\beta A\left(y^{0}\to y^{m}\right)\right)\;Z=\underset{u\in \left\{y^{0}:y^{m}\right\}}{\sum }exp\left(-\beta A\left(u\right)\right),$$

where $\left\{y^{0}:y^{m}\right\}$ indicates the set of trajectories of any length beginning with $y^{0}$ and terminating with $y^{m}$. Given any two endpoints and comparable ε at each step in the trajectory, shorter trajectories will have smaller action and higher probability than longer trajectories. The natural endpoints to consider for these trajectories are the network ground states, as these are the points where the network drives the evolution to converge and where automata theory indicates that the network has “accepted” its inputs (Section 2.2). As is elucidated in Section 2.1, the small-spatial-scale, long-timescale adaptation of an open system should facilitate its large-spatial-scale, short-timescale equilibration with its environment. In the TSMN model we realize this by updating the state transition memory $T_{i}$ to facilitate transitions between network ground states that favor smaller action trajectories.

With these ideas in mind, at each network ground state and *before* the update of the node state-indices, the memory elements $T_{i}$ are updated as

(13) $$T_{i}\left(\gamma_{i}|s_{i}\right)\leftarrow T_{i}\left(\gamma_{i}|s_{i}\right)\cdot \left(1-\alpha \cdot P\left(\gamma_{i}|{\tilde{v}}_{i},s_{i}\right)\right)$$

(14) $$T_{i}\left(\gamma_{i}|s_{i}\right)\leftarrow T_{i}\left(\gamma_{i}|s_{i}\right)+\alpha \cdot P\left(\gamma_{i}|{\tilde{v}}_{i},s_{i}\right),$$

where 0<α<1 is a learning rate parameter, ${\tilde{v}}_{i}$ is the node input at network ground state (i.e., the complement of ${\tilde{y}}_{i}$), and $\gamma_{i}$ indicates that the update is over the set of all possible node outputs. Note that the updates are at a small-spatial-scale because they are different for each node i. The physical motivation for Equation (14) derives from the observation that the negative gradient of the node free energy $F_{i}=-\log Z_{i}$ with respect to $T_{i}\left(y_{i}|s_{i}\right)$ is (see Equation (10))

(15) $$\frac{-\partial F_{i}}{\partial T_{i}\left(y_{i}|s_{i}\right)}=\frac{\partial \log\left(Z_{i}\right)}{\partial T_{i}\left(y_{i}|s_{i}\right)}\sim P\left(y_{i}|v_{i},s_{i}\right).$$

The update rule for the state transition memory $T_{i}$ is therefore a regularized (Equation (13)) gradient descent (Equation (14)) of the free energy of the node over the state transition memory elements conditioned on the current state and input, implemented whenever a network ground state is obtained. Regularization bounds $T_{i}$ such that for repeated application of Equations (13) and (14) for the same ${\tilde{v}}_{i}$ and $s_{i}$, which we might expect if the network experiences periodic inputs, $T_{i}\left({\tilde{y}}_{i}|s_{i}\right)\to 1$ and $T_{i}\left(y_{i}\neq {\tilde{y}}_{i}|s_{i}\right)\to 0$, where ${\tilde{y}}_{i}$ is the output of node i at the *next* network ground state given the current state $s_{i}$. Additionally, to promote short trajectory dynamics, whenever a node receives an edge excitation, the state transition memory of its last output is decayed as,

(16) $$T_{i}\left(y_{i}|s_{i}\right)\leftarrow T_{i}\left(y_{i}|s_{i}\right)\cdot \left(1-\alpha \cdot P\left(y_{i}|v_{i},s_{i}\right)\right),$$

which prevents edge excitations from becoming “stuck” and the long trajectories associated with these local minima. Equation (16) can be thought of as a “metabolic” cost that decays internal organization linked to less-efficient behaviors.

Referring again to the postulates of Section 2.1, Equations (13), (14), and (16) are the large-timescale, small-spatial-scale adaptation algorithm for the TSMN, and Equation (10) and the MCMC algorithm are the short-timescale, large-spatial-scale equilibration algorithm. Scale-integration results from the application of Equations (13) and (14) whenever the MCMC algorithm finds a network ground state. Additionally, because the state transition memory updates happen before the state-index updates at each network ground state, the energy of the network generally increases immediately after the state-index update, reflecting the idea of active equilibration. The effect of this active equilibration is to bias the network away from its current ground state and toward its next ground state.

### 2.6. Inference of Dormant Inputs/Generation of Outputs

Internal nodes infer missing or dormant inputs from connected external nodes using a slight modification of the above methodology. We divide the input vector $v_{i}$ into two components, $v_{i}\to \left(v_{i}^{a},v_{i}^{d}\right),$ where $v_{i}^{a}$ and $v_{i}^{d}$ refer to the active and dormant components of the input to node i. We modify Equation (10) as

(17) $$P\left(y_{i},v_{i}^{d}|v_{i}^{a},s_{i}\right)=\frac{1}{Z_{i}}exp\left(-\beta E_{i}\left(y_{i},v_{i}^{d}|v_{i}^{a},s_{i}\right)\right)\;Z_{i}=\underset{y_{i},v_{i}^{d}}{\sum }exp\left(-\beta E_{i}\left(y_{i},v_{i}^{d}|v_{i}^{a},s_{i}\right)\right),$$

and infer both $y_{i}$ and $v_{i}^{d}$ by sampling Equation (17) over $y_{i}\in \gamma_{i}$ and $v_{i}^{d}\in \left\{0,1\right\}$ for each component of $v_{i}^{d}$. By selection of its output $y_{i}$, node i creates an output to the dormant external nodes which can change the state of those external nodes and the larger environment of which they are a part. After a network has learned to functionalize inputs (see Section 2.1), these outputs can predict dormant inputs and be seen as functions of other, active inputs (see Section 3.2).

### 2.7. Visualization

**Figure 3:**
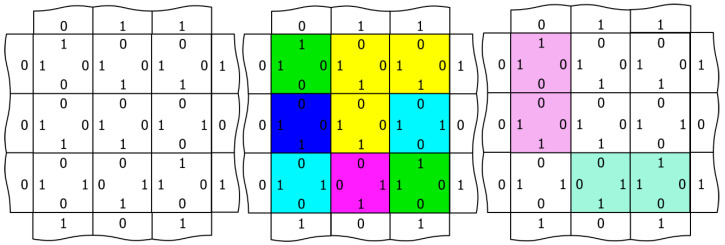
Three different network visualizations. On the left, an array of nine internal nodes with edge codes illustrated as 1 s and 0 s. In the center, each of the 6 node outputs is associated with a different color: red (not shown), green, blue, cyan, magenta, and yellow. On the right, edge-excitations are visualized by coloring the nodes associated with the edge; 0-0 edges are colored light magenta, and 1-1 edges are colored light green.

**Figure 4:**
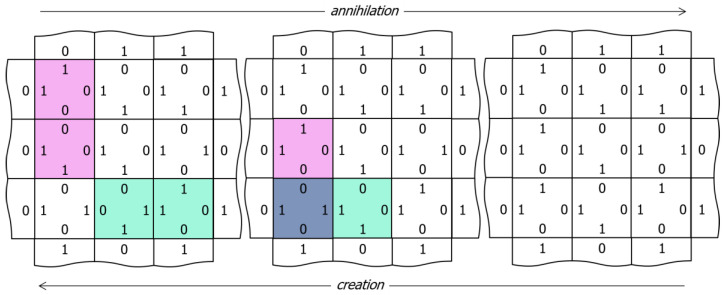
Pair annihilation/creation dynamics. From left to right, complementary excited edges collide and annihilate via a sequence of node output changes. From right to left, a thermal excitation of the lower left node output drives the creation of a pair of excited edges, which diffuse away from the site of their creation via the opposite sequence of node output changes.

Figure 3 illustrates three different ways to visualize the network, which we will employ in the videos of Section 3. Using a 3 × 3 array of nodes, the left-hand image shows the edge codes associated with a particular configuration of node outputs, the central image colors each node according to its output (red, green, blue, cyan, magenta, and yellow), and the right image illustrates edge-excitations among the nodes. Edge-excitations—0-0 or 1-1 code pairs—are illustrated by coloring the nodes attached to the edge in light magenta or light green, respectively. As will become apparent in the videos that follow, the equilibration dynamics of the network are most easily understood as the diffusion, annihilation, and creation of edge-excitations. Figure 4 illustrates this annihilation and creation processes by showing the underlying changes in the edge codes for an example excitation pair.

**Figure 5:**
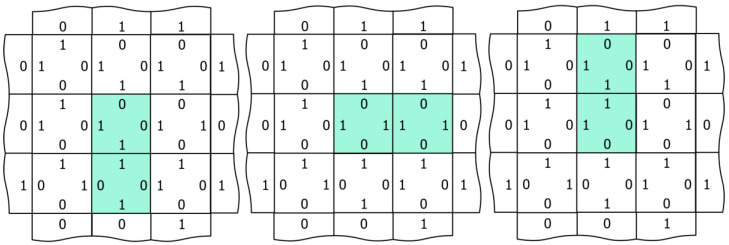
Excited node output degeneracy and edge excitation diffusion in naïve networks. The central node receives divergent inputs (three 1 s and one 0) and must select among three lowest-energy outputs of the same energy illustrated in three separate images. Supposing that the image on the left is the initial configuration, a sampling of the central node, resulting in the configuration of either the central or the right image, means that the edge excitation that was previously associated with the lower central node has “hopped” to another node. In this way, edge excitations diffuse randomly through the network with repeated MCMC sampling of the checkerboard partitions (Figure 1). State transition memory learning lifts this energy degeneracy (Section 2.5), making the dynamics more mechanistic.

As illustrated in Figure 5, the edge excitation dynamics of naïve networks (i.e., networks without state transition memory) are diffusive because the energies associated with node outputs are degenerate: a node with one edge excitation has three equally probable, lowest-energy outputs. Repeated sampling of the network with the MCMC algorithm randomly transfers edge excitations among adjacent nodes. By comparison, nodes receiving non-divergent inputs have a single, lowest-energy output, which makes network ground states stable at low temperatures. To make these same points differently, edge excitations represent an energy barrier separating network ground states. Updates to $T_{i}$ (Equations (13) and (14)) lift the energy degeneracy in Figure 5 and drive the network from diffusive to mechanistic dynamics (see Section 3.2). Decay of $T_{i}$ (Equation (16)) tends to restore the degeneracy.

## 3. Results

We employ the methods of Section 2 to simulate the equilibration dynamics of the TSMN, which we illustrate as a series of videos. We begin with simulations of naïve networks to illustrate diffusive dynamics and the effect of temperature (Section 3.1). These dynamics are characterized by low-energy excitations on a highly degenerate ground state with qualitative resemblance to the “quasiparticles” of Fermi liquid theory. We then simulate networks of increasing complexity that learn a state transition memory as they are exposed to external inputs that are changing periodically in time (Section 3.2). In these networks, a phase transition from diffusive to mechanistic dynamics emerges, after which the inputs are functionalized, the network is scale-integrated, and the equilibration dynamics are active. We show that the TSMN resembles a self-constructed “neural network” after this phase transition.

### 3.1. Networks without Memory

Video 1 presents the equilibration dynamics of a naïve network comprising 400 (20 × 20) internal nodes and periodic boundary conditions. Node outputs are randomized at the start of the simulation and each time the network finds a ground state. Two visualizations—a node output view and an edge-excitation view (see Section 2.7)—of the equilibration dynamics are shown side-by-side. The edge-excitation view of the network shows 1-1 and 0-0 edge excitations (created by the node output randomization at the beginning of each cycle) diffusing, colliding, and annihilating each other. The node output view reveals a patchwork of evolving domains. Although only briefly displayed (a single frame before the network is randomized), the ground states in Video 1 are stable because the simulation temperature is low. Note that the network naturally evolves to find a ground state, which motivated the choice of ground states as the terminating points in the fluctuations considered in Equation (12) and, thereby, the network adaptations of Equations (13) and (14). Video 2 shows the same network as Video 1 at a higher temperature. As in Video 1, edge excitation pairs spontaneously annihilate as they diffuse and collide within the network, but, unlike in Video 1, they also spontaneously emerge from thermal excitations. Hence, there are no long-lived ground states, and equilibrium is achieved when the annihilation and creation rates are equal.

For the remainder of this article, we consider only networks in which the temperature is low enough to inhibit thermal pair creation, which is needed to connect the TSMN to classical computing (Section 4.3). Classical computers operate in a domain where thermal fluctuations are avoided and system states are stable unless intentionally perturbed; hence, their temperature is also low.

Video 3 illustrates the evolution a network like that of Video 1, except that the network contains 2 pairs of randomly placed, complementary external nodes. Each time the network finds a ground state, the “polarity” of the external nodes is inverted (0→1 and 1→0) and the network evolves to find another ground state. The effect of external nodes on the larger network can be thought of in three complementary ways: (1) as constraints that the network must satisfy, (2) as sources and sinks of codes that the network must transport, or (3) as sources of edge excitations that the network must annihilate. Given the large degeneracy of ground states, there are many ways that the network may configure in response to the external inputs to achieve a low energy state. If only a few ground states existed, then the likelihood of escaping local minima to find a low energy configuration would be very small—a problem that is common in some neural network and Ising models. We emphasize that the dynamics of Video 1, Video 2, and Video 3 are entirely diffusive and the equilibration times are correspondingly long because there is no state transition memory and no learning to break the node output energy degeneracy of Figure 5.

### 3.2. Networks with Memory

Video 4 repeats the simulation of Video 3 but includes learning of the state transition memory. The initialization of the state transition memory is unbiased, $T_{i}\left(y_{i}|s_{i}\right)=0$ for all i, $y_{i}$, and $s_{i}$, so the network begins in a naïve state. As in Video 3, in the early stages of the simulation the equilibration dynamics are slow and diffusive, but, as the state transition memory is learned, a phase transition emerges, and the equilibration dynamics become fast and mechanistic. To state this another way, the trajectories between network ground state equilibrations change from being random and long to being regular and short. Additionally, before the phase transition, the network dynamics cannot be described as a function, but after the phase transition the network dynamics can be described by a function—namely, as a network of (nearly) deterministic state machines.

**Figure 6:**
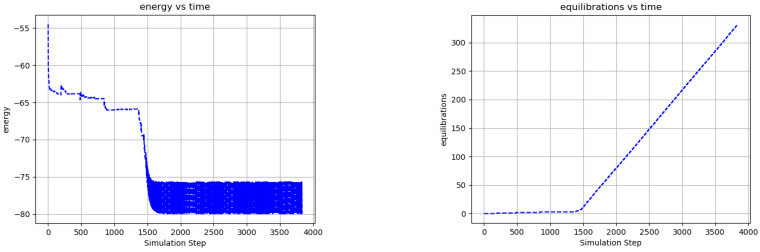
Network statistics for Video 4 illustrating a phase transition. Average node energy (**left**) and number of ground state equilibrations (**right**) versus simulation step. A phase transition emerges after approximately 1500 simulation steps and 8 ground state equilibrations, marking the transition from diffusive to mechanistic dynamics. The emergence of the state transition memory is illustrated in the reduction in energy as the simulation progresses. A spike in the energy occurs after each ground state equilibration, owing to the introduction of new edge excitations from the external nodes and to the change in the state transition memory. These energy spikes are dissipated as the edge excitations propagate through the network and annihilate each other.

Figure 6 shows the evolution of the average node energy and the number of ground state equilibrations vs. simulation step for the network of Video 4. The rapid changes in the node energy and equilibration rate around step 1500 indicate a phase transition from diffusive to mechanistic dynamics. In this simple system, the phase transition emerges after approximately eight equilibrations. A spike in the energy occurs after each ground state equilibration, due both to an influx of new edge excitations from the external nodes and to changes in the internal network state. At each new network ground state, an updated state-index $s_{i}$ addresses a new memory element $T_{i}$, which biases each node output toward its output at the *next* network ground state, and which generally *increases* the energy of the current node output (see Section 2.5). The size of these energy spikes increases as the network learns (because $T_{i}$ increases) but the overall network energy decreases at the same time. In other words, the effect of $T_{i}$ is to place the nodes in a metastable configuration after each ground state transition that facilitates the next ground state transition while also reducing the energy of the overall system. This storage and release of energy to equilibrate with an environment corresponds to the active equilibration postulate of Section 2.1.

After the phase transition from diffusive to mechanistic dynamics, the TSMN displays input functionalization (see Section 2.1), which we can illustrate by turning off inputs from the external nodes in the network, and allowing the internal nodes connected to these dormant external nodes to infer the missing inputs (Section 2.6). In Video 5, we explore the effects of dormant external nodes and the weighting parameter σ on the same type of network as in Video 4. As is evident in Video 5, the TSMN has functionalized its inputs after the phase transition because it can infer missing inputs from dormant external nodes and provide edge excitations as outputs to the dormant external nodes. The size of σ affects the detailed dynamics significantly by specifying the degree to which the dynamics are internally generated by the state transition memory (i.e., $T_{i}$) versus externally stimulated by the active inputs and communicated by the internal node interactions (i.e., $H_{i}$).

**Figure 7:**
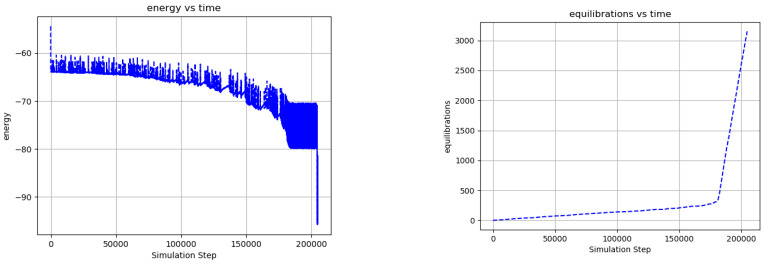
Network statistics of Video 6, illustrating a phase transition. Average node energy (**left**) and number of ground state equilibrations (**right**) versus simulation step. A phase transition emerges after approximately 150,000 simulation steps and 300 ground state equilibrations, marking the transition from diffusive to mechanistic dynamics. Each spike in the network energy corresponds to the introduction of new edge excitations by the external nodes, and a change in the state transition memory of the internal nodes after a ground state is achieved. The emergence of the state transition memory is illustrated by the reduction in the overall network energy and an increase in energy spikes as the simulation progresses. Although state transition memory decay (Equation (16)) is ongoing throughout the simulation, a relatively long period without ground state equilibrations around step 130,000, where the network energy is increasing, highlights this decay and the escape from a local minimum. The large change in energy at the end of the simulation is generated by a change in the weighting of the state transition memory from σ=1 to σ=2.

Video 6 shows the evolution of a network including 12 external nodes that change polarity with 3 different periods, and a corresponding state depth d=3 is assumed for the state transition memory. Figure 7 captures network statics corresponding to Video 6. Because the size of the state transition memory scales as a power law with exponent d, the network of Video 6 undergoes a much larger number of ground state equilibrations before undergoing a phase transition, as compared to Video 4 (compare Figure 6 and Figure 7). As in Video 5, various configurations of external node dormancy and state transition memory weighting are explored to illustrate input functionalization. We note that some stochasticity in the network dynamics remains even after the phase transition and that this stochasticity increases as the number of dormant inputs increases. The complexity of the network dynamics in Video 6 when all the external nodes are made dormant and σ=1 suggests a kind of “dreaming” or “thinking” about what has been experienced in the past—an imperfect, somewhat stochastic version of that past. When the state transition memory weighting is increased to σ=2, the network displays rapid, mechanistic dynamics that are independent of external node inputs, suggesting the ability to “think ahead” of the dynamics that initially produced it.

Video 7 is an edge-excitation view of a somewhat larger network after the phase transition, in which static nodes are colored gray to emphasize the structure of the network. Interestingly, the spatial structure of the evolved network looks like a neural network in which external nodes are connected by axon/dendrite-like channels communicating via spike-like edge excitations. There is even the suggestion of “integration” of these spikes before a neuron “fire”. When the external nodes are made dormant, the network dynamics are more stochastic, but the underlying spatial organization remains. We note that the ground state degeneracy and stochasticity inherent in the evolution of the TSMN means that Video 7 illustrates one among many “phenotypes” that might have emerged.

## 4. Discussion

In this section, we discuss the limitations of the TSMN (Section 4.1), speculate on a new approach—thermodynamic computing—to address these limitations (Section 4.2), and offer an interpretation of classical computing considering the ideas presented herein (Section 4.3).

### 4.1. Limitations of the TSMN

Generality—The TSMN has been carefully constructed such that the equilibration and adaptation algorithms of the network model are computable (see Section 2.3 and Section 2.5). In particular, the ability to sample the node outputs independently (Equations (7) and (10)) is critical. We emphasize that these algorithms, while motivated by generic thermodynamic considerations, are in no way general—they work specifically for the purposes of the TSMN. In this respect, the TSMN is just one among many machine learning methods, each employing specialized algorithms of equilibration and adaptation (or, equivalently, optimization and learning) directed toward a particular task. On the other hand, the equilibration and adaptation of natural systems are seemingly not constrained by their “computability” in the same way.

Scalability—In general, the number of possible network configurations in the TSMN grows exponentially with the number of nodes, connections, edge codes, and state depth. For example, even in the 400-node networks shown above, the total number of possible network outputs (6^400^ ≅ 10^311^) is staggering. The effect of this very large configuration space on the computability of the TSMN is different, however, depending on whether a phase transition in the dynamics has emerged. Before the diffusive-to-mechanistic phase transition, the computational burden is large because the diffusive search for network ground states must, in principle, address the entire configuration space, and because the recursive structure of the TSMN makes the search non-deterministic. Powerful machine learning models often avoid such difficulties by eliminating recursion in the network [22], but computing resources needed to train state-of-the-art machine learning systems are still doubling every 3.4 months according to a recent report [1]. We suspect that success in finding the ground states in the examples above hinges on the relatively short diffusion distances in these small networks and the availability of many ground states ((3/2)^400^ ≅ 10^70^). After the phase transition, however, the network dynamics become mechanistic, and the combinatorics of the configuration space are largely irrelevant. In essence, the learning process finds and memorizes an efficient short-cut through the large combinatorial space (possible because the inputs driving it are periodic and predictable). In summary, the equilibration algorithm of the TSMN scales poorly in the learning phase, but scales well in the mechanistic phase. The same can be said of other machine learning models and computing in general.

Temporality—The dynamics of the TSMN between ground state equilibrations are nearly (Equation (16)) reversible fluctuations (Equation (10)) that do not affect the state of the network. Irreversibility in the TSMN is introduced via the updates to the state indices (Equation (9)) and learning of the state transition memory (Equations (13) and (14)) after all edge-excitations are annihilated and a network ground state is obtained. After the phase transition, this irreversibility manifests as mechanistic dynamics. Hence, the TSMN connects energy dissipation to irreversibility to the emergence of mechanistic dynamics. Additionally, the same equilibration algorithm drives both the diffusive and the mechanistic dynamics in the TSMN—there is no need to invoke separate dynamical principles a priori. Hence, the TSMN makes a clear connection between thermodynamics and temporality, but, unlike natural systems, the TSMN model is still a collection of specialized methods contrived to work well on a computer.

### 4.2. Thermodynamic Computing

Thermodynamic Computers (TCs) are technological systems built to address the limitations of Section 4.1. With others, we have previously described the motivation and vision for TCs [2,3,4]. The key hypothesis is that natural, multi-scale, thermodynamic self-organization can be an integral part of computing system hardware. In this section, we further suppose that TCs will address the postulates that motivated the TSMN (Section 2.1) and comment on the published efforts that have a bearing on these hypotheses (summarized in Table 1).

In a remarkably prescient but also very difficult exposition, Pask [23] describes most of the core concepts of a thermodynamic computer with experiments on the evolution of wires in ferrous sulfate solutions in response to external electrical potentials. Pask is concerned with the evolution of structures in physical systems and their correspondence to the evolution of concepts through “thinking”. He proposes several requirements for such systems, including continuous state change, conservation of transported quantities (charge), dynamic equilibrium with an environment, organizational degeneracy, extended excited states, thermodynamic openness, component plasticity, decay of structure, and non-mechanistic behavior. In describing resistor networks with a negative temperature coefficient of resistance, he also anticipates some of the functionality of the resistive memory elements described in the examples below.

Jun & Hübler [24] have shown collections of several hundred small steel balls in a dish of castor oil, organizing in response to an electric field between the center and the periphery of the dish. From various initial placements, when the electric field is applied, the balls move to form branching networks. When one of these branches succeeds in creating a strong electrical connection between the electrodes, a phase transition in the ball dynamics is observed. Comparing this to the idea of two-component scale-integration (Section 2.1), the movement and organization of the balls plays the role of the small-spatial-scale, large-timescale adaptation, and the electron transport through these balls plays the role of the large-spatial-scale, short-timescale equilibration. The two are naturally integrated by the charges that collect on and are transported through the steel balls, which organize the collection of bearings as the system “learns”, resulting in a phase transition.

Thompson [25] demonstrate on an FPGA the evolution of a circuit that discriminates between two input frequencies (“tones”), which are much lower than the characteristic frequency of communication within the FPGA. Using an external computer to evaluate a fitness function over the FPGA output, and a genetic algorithm to sample FPGA configurations creating this output, the system evolves the ability to effectively discriminate between the tones using the underlying physics of the FPGA substrate. The method employs two timescales of evolution—the rapid dynamics of the entire FPGA and the slower, local adaptation of the genetic algorithm. Additionally, the system can functionalize its inputs and generate outputs corresponding to dormant inputs on which it was trained. Remarkably, the evolved “solution” employs physics that is entirely outside the design domain of the FPGA when used in a conventional sense.

Sillin [26] models a complex network of Ag nanowires with Ag_2_S junctions (“atomic switch networks”) electrically biased by external electrodes in contact with a subset of the nanowires. These models, which correspond well with experimental results, show complex network dynamics driven by the coupling between electron conduction through the network and Ag^+^ ion mobility within the Ag_2_S junctions. State-dependent memory effects captured within the Ag_2_S junctions are also evident. In related work, some of these same authors show memory and scale integration [27] as well as dynamical phase transitions [28] in networks of Ag nanowires with polymer junctions between nanowires. Others show similar effects in networks of conducting nanoparticles near a percolation threshold [29,30]. In the language of the TSMN, low-energy configurations in the atomic switch networks are those that find strong conducting pathways through the network that connects the external electrodes [31]. The finding of such pathways creates a large flow of energy through the network, some portion of which changes the state of the Ag_2_S junctions. Although these networks have been modeled for use as reservoir computers in temporal prediction tasks [32,33], they have not been shown to directly infer or predict a missing input. Perhaps a missing element within these systems as compared to the TSMN model is the ability to access or store energy internally and, thereby, to create the active dynamics needed to functionalize inputs. Another noteworthy effort [34] illustrates engineered, multiscale adaptation in networks of diffusion dominated (“neurons”) and drift dominated (“synapses”) memristive devices.

In other examples employing the physics of the computing substrate to accomplish a task, an engineer defines a problem by encoding certain parameters (e.g., weights or couplings) into (real or simulated) hardware, and the equilibration dynamics of the hardware solve the problem by finding a low energy configuration. Often, the problems are posed as an NP combinatorial problem, such as k-SAT, max-cut, or knapsack, which can be mapped to an Ising model [35]. Examples include Ising machines realized in CMOS annealers [36,37,38], coupled oscillator networks [39,40,41], noisy memristor crossbars [42], and quantum annealers [43]. While emphasizing the use of probabilistic magnetic bits (“p-bits”) as key enabling elements, [44,45,46,47] it is possible to extend these ideas to applications of invertible logic and integer factorization in probabilistic analog hardware, digital hardware, and simulation. Lee [48] explores more complex magnetic systems, which they call “magnetic thermodynamic cores”. The key task for all these Ising/Boltzmann Machines is to rapidly equilibrate toward the ground state of an Ising model given a network of coupling coefficients corresponding to a problem of interest. All these examples suffer from challenges with escaping local minima, device non-uniformity, and scaling to large networks. Among these approaches, we note the “device/circuit-level” approaches where the physics is relatively transparent, low-level hardware “computational” approaches where it is obscured, and simulations where it is entirely modeled. Hence, these Ising machines illustrate the tradeoffs associated with computation-based approaches, physics-based approaches, and the messy area between them. While all these efforts share motivation with the TSMN, they do not address the postulates of scale integration and input functionalization.

Traversa and Di Ventra [49] demonstrate the ability to evolve solutions to NP combinatorial problems with polynomial resources using a network of “self-organizing logic gates” called a “digital memcomputing machine.” Like the examples in the previous paragraph, the problem to be solved is encoded by an engineer into the topology of a network, which the authors describe as the “information overhead” of the network. These self-organizing logic gates do not distinguish between inputs and outputs in the traditional sense; as networks of these gates interact, conflicts between logical assertions at each gate terminal are resolved, and an equilibrium state emerges that represents the solution to the problem encoded in the network topology. These same authors [50] describe the function of these networks in terms of dynamical systems theory, stress the intrinsic parallelism that it affords, and interpret its ability to solve NP problems using polynomial resources in terms of instantons that rapidly descend a hierarchy of critical points. Di Ventra et al. [51] generalize these ideas with a theory of topological symmetry breaking in dynamical systems. The authors argue that digital mem-computing machines can be realized in hardware as well as simulated as dynamical systems on a classical computing platform. The digital mem-computing machine approach addresses the same problem sets as in the previous paragraph and, like them, does not address all the postulates listed above. However, the mem-computing approach details important concepts that are necessary for the realization of a thermodynamic computer, including input-output equivalence, collective dynamics in classical systems, spatial non-locality stored in local memory adaptation, the role of conservation (Kirchhoff’s) laws, the emergence of instanton dynamics, the role of engineered information overhead in defining a problem, and the need for intrinsic parallelism.

Given the central role of thermodynamics, chemical systems are a natural focus for the development of thermodynamic computing systems and concepts. Turing [52] perhaps anticipated this possibility in his work on pattern formation from homogeneous chemical systems. More recently, Dueñas-Díez and Pérez-Mercader [53] have shown that chemical systems can recognize words within context-sensitive languages, where the symbols in the language correspond to the introduction of aliquots into a simple, one-pot reactor. With this example, it is possible to consider any chemical reaction as a kind of thermodynamic state machine, with reactants playing the role of the self-organizing node interactions and catalysts playing the role of a state transition memory facilitating the approach to equilibrium. If the massive “parallelism” afforded by an Avogadro’s number of reactants can be configured into interacting “cells”, each with a much smaller number of molecules, large networks of thermodynamic automata not unlike the TSMN might be achieved. Researchers from this same group [54] have recently demonstrated important steps in this direction in more complex photochemical systems, in which cell-like vesicles—“chemical protocells”—spontaneously assemble, collapse, move, and interact with each other while carrying internal chemistry unique to their history. The various evolutionary processes in these systems naturally integrate scales and sample diverse configurations. The authors are motivated to understand the origins of life, but the connections to computing systems that organize themselves thermodynamically are a natural extension.

The approaches summarized above suggest that TCs can be constructed from hybrids of diverse electronic, electro-ionic, electro-mechanical, chemical, and electrochemical components. The integration of their diverse spatial and temporal scales also suggests an ability to create complex, evolving, self-organizing, technological systems such as the TCs envisioned here.

### 4.3. Classical Computing

Every classical computer is realized on a physical substrate (e.g., semiconductor chip) in which the transitions between states are driven by a changing energy landscape. With each clock cycle, a classical computer equilibrates to a new ground state (typically referred to as the computer’s “state”), which, like the TSMN, can be associated with the acceptance of a “word” in the “language” of the computer. Like the TSMN, a classical computer has a very large number of ground states, transitions between them are driven by the transport of a conserved quantity (electric charge, typically), many different configurations can achieve the same input–output function, and the dynamics are active. Unlike the TSMN, however, the fluctuation dynamics in a classical computer are ignored: only the sequences of ground state transitions of a classical computer are considered as its dynamics. Additionally, for a classical computer, scale-integration is the result of painstaking hardware and software engineering that effectively links enormous numbers of (very small) transistors to create an output for rapid consumption by a (very large) human. Typically, this scale-integration is facilitated via a hierarchy of abstraction, or “stack”, corresponding to various scales in the integration process. In other words, scale-integration in a classical computer resides within the evolutionary capacity of engineers working across many scales. With this in mind, we can draw further correspondence between the TSMN model and a classical computing system that includes its engineers. In particular, the phase transition in the TSMN dynamics corresponds to engineers obtaining a desired input/output function after an evolutionary, engineering process in which the design inputs are functionalized. In summary, classical computing works because engineers are available to perform the evolution required to integrate scales and functionalize inputs. Pablo Picasso was once quoted as saying “computers are useless. They can only give you answers” [55]. In the context argued here, we speculate that Picasso was commenting on the incompleteness of computers—namely, their inability to perform the evolutionary processes needed to solve problems or create works of art.

### 4.4. Thermodynamicalism

We speculate here on a different philosophical perspective that summarizes the discussion of Section 4.1, Section 4.2 and Section 4.3 and builds upon the postulates of Section 2.1. We call this perspective *Thermodynamicalism* and distinguish it from the perspective known as *Computationalism*, which dominates computing, engineering, and science today.

*Thermodynamicalism* is the philosophy that open thermodynamic systems equilibrating with external potentials will *self-organize* into *functional systems* that are *computable* after they have organized, but that the equilibrating, self-organizing process itself, which we call *thermodynamic evolution*, is not computable *in general*.

By *self-organization*, we mean a dynamic process in which a system can store energy, act upon its environment, and change its internal organization, such that equilibration with external potentials is more efficient and entropy production in the environment is sustained, even as the environment changes. By *functional systems*, we mean systems linking inputs and outputs with some internal degrees of freedom that can be described by an algorithm or mathematical function. By *computable*, we mean that the dynamics of the system can be simulated using pragmatic computing resources. The qualifier *in general* allows that certain model systems—e.g., the TSMN—have highly specialized, computable models of their evolution, while stipulating that the evolution of real physical systems of any meaningful scale is not computable. Stated differently, thermodynamicalism says variously that there is no general equilibration algorithm, no general algorithm for the second law of thermodynamics, or no general algorithm for evolution. These statements reflect the limitations of the TSMN (Section 4.1), motivate the development of thermodynamic computing (Section 4.2), and explain the incompleteness of classical computing (Section 4.3). In a nutshell, thermodynamicalism supposes that algorithms, which are the unexplained foundation of computationalism, are the artifacts of a thermodynamic, evolutionary process that is not computable in general.

## 5. Conclusions

Employing four postulates concerning open thermodynamic systems—transport driven self-organization, scale integration, input functionalization, and active equilibration—we have elaborated a new methodology for a new network- and machine-learning model, the Thermodynamic State Machine Network. The TSMN is a network of probabilistic automata, exchanging conserved codes over unweighted bi-directional edges, equilibrating according to Boltzmann statistics, and is characterized by a large degeneracy of ground states separated by particle-like excitations. The TSMN includes a state transition memory that learns to minimize the action of fluctuation pathways between network ground states. When exposed to periodic external inputs, the TSMN undergoes a phase transition from diffusive to mechanistic dynamics. After the phase transition, the TSMN can infer or predict missing inputs, and spontaneously generate internal dynamics representative of inputs it has functionalized. Additionally, the dynamics of the TSMN resemble those of spiking neural networks, although no such structures were incorporated into the initial model. We consider the postulates used to construct the TSMN in the context of thermodynamic computing systems and compare similarly motivated experimental efforts in the literature. We suggest that thermodynamic computing can address the fundamental limitations of the TSMN, other machine learning approaches, and classical computing in general. Given prior art, we can see no fundamental reason that thermodynamic computing systems cannot be developed. We offer speculation on a different philosophical perspective, thermodynamicalism, to address the challenges of computationalism.

## Figures and Tables

**Video 1:**
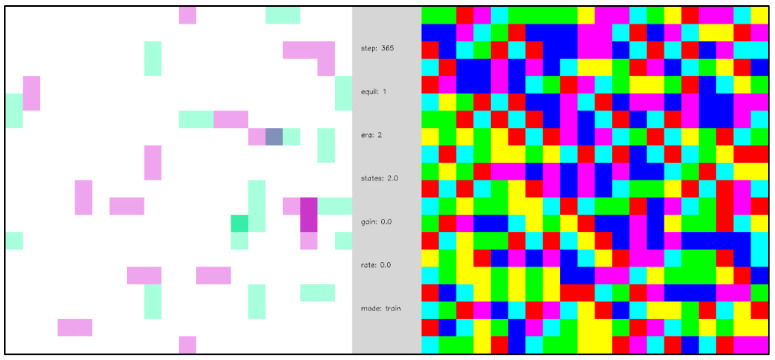
Closed-system equilibration at a low temperature. A naïve network of 400 internal nodes with periodic boundary conditions and inverse temperature β=16. The node outputs are randomized initially and each time the network reaches a ground state. On the left-hand side, complementary edge excitations, created when the network is randomized, annihilate as the network evolves toward a ground state. On the right-hand side, the node outputs organize into local domains that change as the network evolves toward a ground state. This simulation shows the evolution to 4 different ground states, each having the same energy. Video S1 and at https://youtu.be/bqn0qdvo4Kc (uploaded 5 October 2021).

**Video 2:**
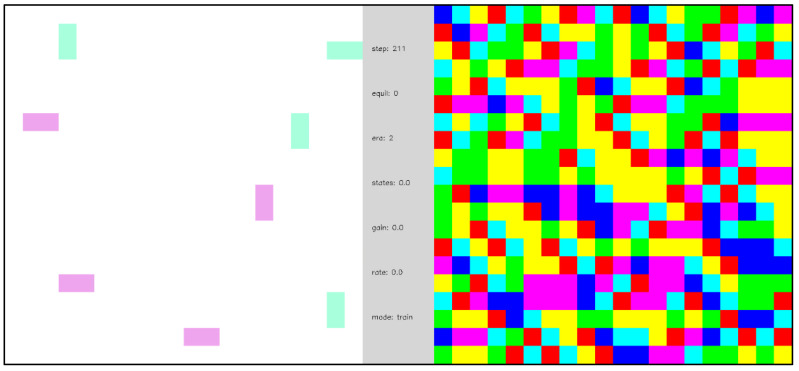
Closed-system equilibration at a high temperature. A naïve network of 400 randomly initialized internal nodes with periodic boundary conditions and inverse temperature β=4.5. On the left-hand side, complementary edge excitations are both annihilated by collisions and created by thermal excitations. On the right-hand side, the local node output domains continuously evolve. Unlike in Video 1, thermal excitations prevent the formation of stable ground states. Video S2 and at https://youtu.be/cpt4Yi_3hEE (uploaded 5 October 2021).

**Video 3:**
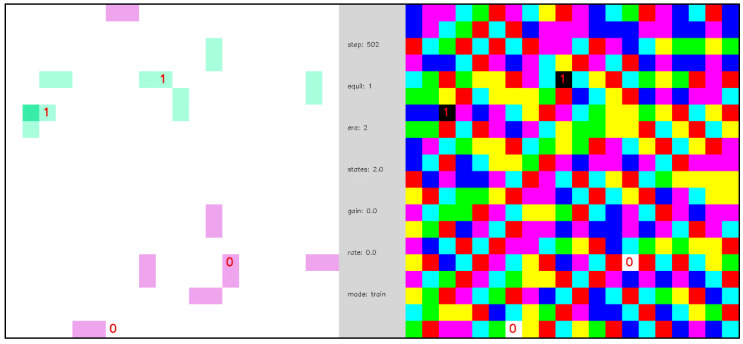
Open-system equilibration with periodic external inputs. A naïve network of 396 internal nodes and 2 pairs of randomly placed, complementary external nodes with periodic boundary conditions and inverse temperature β=16. External nodes are labeled as 0 or 1 to indicate their polarity. Each time the network reaches a ground state the polarity of the external nodes is reversed. A total of four different ground states are found in this simulation. Ground states are stable because the temperature is low. Video S3 and at https://youtu.be/SQLzvfKfLgs (uploaded 5 October 2021).

**Video 4:**
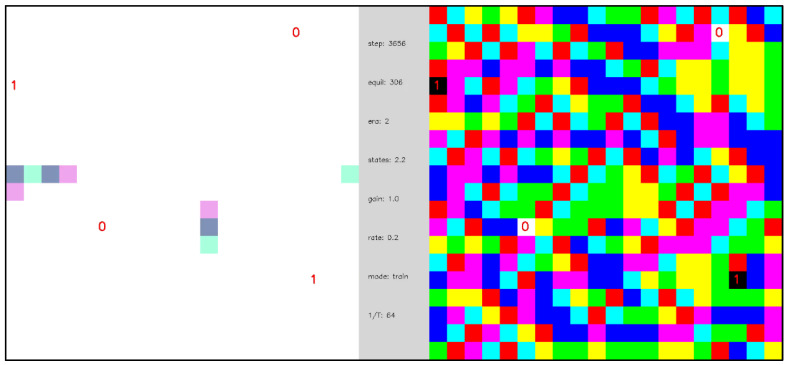
Open system with periodic external inputs, illustrating a dynamical phase transition. A network of 396 internal nodes and 2 pairs of complementary external nodes with periodic boundary conditions, β=16, and state transition learning with state depth d=1. External nodes are labeled as 0 or 1 to indicate their polarity. Each time the network reaches a ground state the polarity of the external nodes is reversed. In the first segment of the video, the first two ground state equilibrations of the network are shown, and the dynamics are diffusive and slow. In the second segment of the video, after a gap of 300 additional ground state equilibrations (indicated as gray-colored frames in the video) in which the state transition memory is learned, the dynamics between ground states become fast and mechanistic. The central panel displays simulation parameters including the number of simulation steps (“steps”), the number of ground state equilibrations (“equil”), the average number of node states realized (“states”), the transition memory weighting parameter σ (“gain”), and the learning rate α (“rate”). Video S4 and at https://youtu.be/7H0AdQ1PxsY (uploaded 5 October 2021).

**Video 5:**
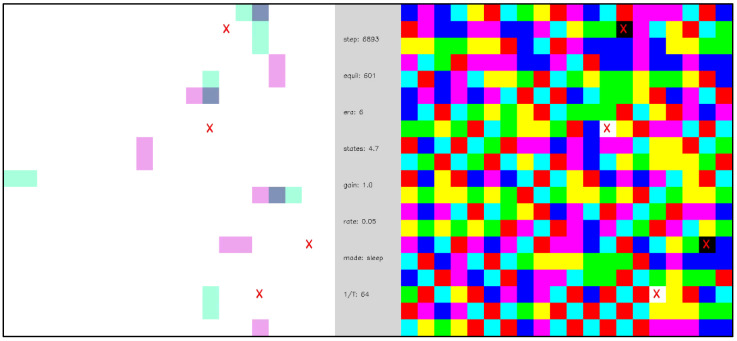
Open system with periodic external inputs, illustrating a dynamical phase transition and input functionalization. A network of 396 internal nodes and 2 pairs of complementary external nodes with periodic boundary conditions, β=16, and state transition learning with state depth d=1. External nodes are labeled as 0 or 1 to indicate their polarity. Each time the network reaches a ground state the polarity of the external nodes is reversed. The first two ground state equilibrations are shown, and the network dynamics are diffusive and slow. After a gap of 400 ground state equilibrations, the equilibration dynamics are fast and mechanistic with σ=1 (denoted as “gain” in the central panel). From steps 5663–5960, two external nodes are made dormant—denoted by the “X” labels—and the dynamics remain similar. From steps 5961–6441, σ=0.5 and the dynamics become feedforward propagation of edge excitations from active to dormant external nodes. From steps 6442–6552, σ=2 and the internal nodes spontaneously create edge excitation pairs that rapidly annihilate with other pairs. From step 6553, all external nodes become dormant and the network “makes its own dynamics” for σ=1 and σ=2. Video S5 and at https://youtu.be/T9tnIn-enFk (uploaded 5 October 2021).

**Video 6:**
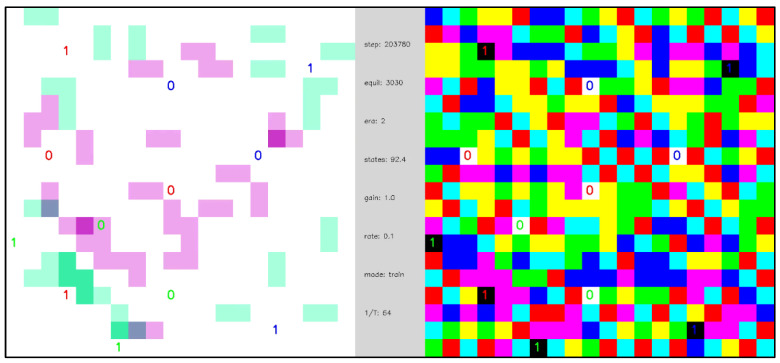
Open system with multi-periodic external inputs, illustrating a dynamical phase transition and input functionalization. A network of 388 internal nodes and 3 groups of 2 pairs of complementary external nodes with β=16 and d=3. External nodes are labeled as 0 or 1 to indicate their polarity. Each external node group changes polarity with a different period of ground state equilibrations—the three external node groups are distinguished by the color (red, blue, green) of the text indicating the polarity of the node. Each time the network reaches a ground state the polarities of the external nodes are updated according to their period. The first two ground state equilibrations are shown, and the network dynamics are diffusive and slow. After a gap of 3000 ground state equilibrations (gray frames), the equilibration dynamics are fast and mechanistic. From steps 203545–203793 the network dynamics are visualized with σ=1 (denoted as “gain” in the central panel). From steps 203794–204092, six external nodes are made dormant (two per group)—denoted by the “X” labels—and the dynamics remain similar. From steps 204093–204471, all 12 external nodes are made dormant, and the network maintains similar dynamics, but with substantially greater stochasticity in the trajectories of the particles. From steps 204093–204605, the transition memory weighting is increased to σ=2, and six external nodes are made dormant. The network spontaneously generates internal excitation pairs that rapidly annihilate to attain ground states. From step 204606 forward, all external nodes become dormant and the network effectively “makes its own dynamics”. Video S6 and at https://youtu.be/TypFJ5NQJZM (uploaded 5 October 2021).

**Video 7:**
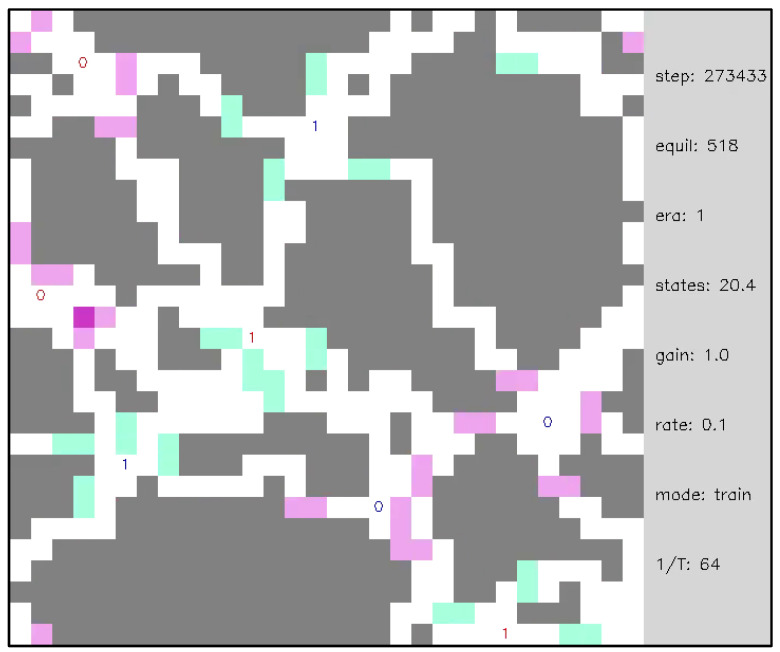
Open system with multi-periodic external inputs, illustrating neural network structure after a dynamical phase transition. A network of 892 internal nodes and 2 groups of 2 pairs of complementary external nodes with β=16 and d=2. External nodes are labeled as 0 or 1 to indicate their polarity. Each external node group changes polarity with a different period of ground state equilibrations—the two external node groups are distinguished by the color (red, blue) of the text, indicating the polarity of the node. Each time the network reaches a ground state, the polarities of the external nodes are updated according to their period. After a gap of roughly 100 ground state equilibrations (gray frames) in which the state transition memory is learned, the equilibration dynamics are fast and mechanistic. Internal nodes that maintain fixed outputs are colored gray to emphasize the underlying spatial structure of the evolved network. From steps 273,211–273,695 the external nodes are active and from 273,696–274,650 the external nodes are dormant. External nodes resemble neurons connected by axon/dendrite-like channels communicating spike-like edge excitations. Video S7 and at https://youtu.be/IGEdvnJM4oA (uploaded 31 October 2021).

**Table 1:** Comparison of Thermodynamic Computing-related works.

	Natural Equilibration	Transport Driven Self Organization	Input Functionalization	Scale Integration	Active Dynamics
Ferrous sulphate electrochemistry (e.g., Pask 1958)	Yes	Yes	Yes	Yes	No
Steel bearing electromigration (e.g., Jun 2015)	Yes	Yes	Yes	Yes	No
FPGA evolution (e.g., Thompson 1996)	No	Maybe	Yes	Yes	Yes
Atomic Switch Networks (e.g., Sillin 2013)	Yes	Yes	Yes	Yes	No
Ising Machines (see text)	Yes, in some cases	No	No	No	Yes
Memcomputing Machines (e.g., Traversa 2017)	Yes, in principle	Maybe	No	Yes	Yes
Chemical Protocells (e.g., Pearce 2021)	Yes	Partially	Yes	Yes	Yes
Machine Learning (e.g., TSMN model)	No	Yes	Yes	Yes	Yes

**Figure A1:**
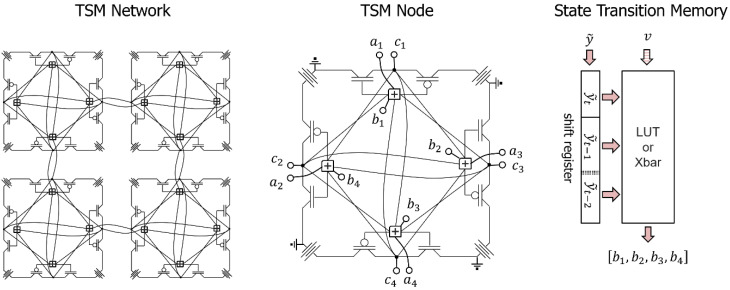
Conceptual circuit implementation of the TSMN. Each node is an array of four inverters that generate the output of the node, $y=\left[c_{1},c_{2},c_{3},c_{4}\right]$. The inputs to each inverter are a sum of the other inverter outputs in the node and the inputs $\left[a_{1},a_{2},a_{3},a_{4}\right]$ and from an inverter in another node along a corresponding edge. The circuit fosters a competition within the node to achieve a non-divergent output that complements its inputs. The state transition memory is a lookup table or crossbar array that outputs a bias vector $\left[b_{1},b_{2},b_{3},b_{4}\right]$ to the summing junctions, indexed by a shift register storing the node state (the last d outputs $\tilde{y}$ at a network ground state) and trained each time a network ground state is obtained. The state transition memory is decayed whenever inputs v create an excited state. Not shown are a network signal indicating that a ground state has been reached and a memory decay concept. Details concerning the training of the bias vectors and the feasibility of the suggested implementation are still unknown.

## Data Availability

Not applicable.

## Supplementary Materials

The following supporting information can be downloaded at: https://www.mdpi.com/article/10.3390/e24060744/s1; Video S1: Closed-system equilibration at a low temperature, Video S2: Closed-system equilibration at a high temperature, Video S3: Open-system equilibration with periodic external inputs, Video S4: Open system with periodic external inputs, illustrating a dynamical phase transition, Video S5: Open system with periodic external inputs, illustrating a dynamical phase transition and input functionalization, Video S6: Open system with multi-periodic external inputs, illustrating a dynamical phase transition and input functionalization, Video S7: Open system with multi-periodic external inputs, illustrating neural network structure after a dynamical phase transition.

## Appendix A. TSMN Circuit Model

Figure A1 is a conceptual circuit for an implementation of a TSMN, showing a network, node, and state transition memory. Each node is an array of four inverters, where each inverter input is the sum of inputs from the three other inverters in the node, from the inverter of a connected node, and from a state transition memory. The idea is to create a competition among the inverters to satisfy the requirements of non-divergence, complementary edge codes, and bias learned through prior state transitions. We note that Figure A1 is not a computational substrate for the mathematical expressions above, rather, it is a circuit that captures the physical ideas that motivated the TSMN. Although Figure A1 illustrates typical CMOS inverters, stochastic inverters, such as those employing magnetic tunnel junctions [47], may be a better choice to achieve the diffusive behavior of the edge excitations shown in Figure 5.
